# MYD88^WT^CXCR4^MUT^华氏巨球蛋白血症1例

**DOI:** 10.3760/cma.j.cn121090-20231219-00326

**Published:** 2024-06

**Authors:** 水个 杨, 慕晨 张, 杰 熊, 婷 黄, 香琴 翁, 黎 王, 维莅 赵

**Affiliations:** 上海交通大学医学院附属瑞金医院血液科，医学基因组学国家重点实验室，上海血液学研究所，上海 200025 Ruijin Hospital, Shanghai Jiao Tong University School of Medicine, State Key Laboratory of Medical Genomics, Shanghai Institute of Hematology, Shanghai 200025, China

患者，女，76岁，2021年3月起多次无明显诱因出现腹泻，大便不成形，黄色水样便为主，7～8次/d，伴有腹胀，无明显腹痛，无发热，就诊于上海交通大学医学院附属瑞金医院消化科门诊，增强CT检查提示部分小肠壁增厚水肿。给予止泻、调节肠道菌群等治疗后好转。既往高血压病史10年。2021年4月7日患者因“面、颈部疱疹2 d”于上海交通大学医学院附属瑞金医院皮肤科住院。查体：贫血貌，头面部左侧散在分布多个绿豆大小红斑，部分红斑上覆水疱。血常规：WBC 4.04×10^9^/L、HGB 86 g/L、PLT 211×10^9^/L。生化常规：总蛋白90 g/L、白蛋白30 g/L、血钾2.99 mmol/L、LDH 128 IU/L、红细胞沉降率121 mm/1h。免疫球蛋白：IgG 10.09 g/L、IgA 2.12 g/L、IgM 51.23 g/L。心电图大致正常。心脏超声：轻度三尖瓣关闭不全，肺动脉高压，收缩压41 mmHg（1 mmHg＝0.133 kPa），即时心率62次/min。腹部超声未见明显异常。住院期间再次出现腹泻，给予止泻、调节肠道菌群、补液支持等治疗，腹泻症状无好转。因球蛋白明显升高转入上海交通大学医学院附属瑞金医院血液科。血清蛋白电泳：在γ区可见M峰（占24.51％）；血免疫固定电泳：检出单克隆免疫球蛋白（M蛋白）为IgM、κ型。骨髓象：增生活跃，粒红比值减低，粒系增生活跃，红系增生活跃，以中晚幼红细胞为主，成熟红细胞可见缗钱状排列，巨核系增生活跃，血小板散在或成簇可见，成熟淋巴细胞比例偏高（占35％），可见少量幼稚淋巴细胞，部分可见浆样分化，成熟浆细胞占5.5％，考虑淋巴浆细胞淋巴瘤。骨髓免疫分型：可见3.4％的单克隆浆细胞，免疫表型为CD138^+^CD38^+^CD19^+^CD56^−^CD117^−^CD27^+^CD81^+^CD45^dim^cLambda^−^cKappa^+^，胞质Kappa限制性表达。另外可见5.6％的成熟小B淋巴细胞，免疫表型为CD19^+^CD5^−^CD10^−^CD23p^+^CD22^+^CD79b^+^CD20^+^FMC7^+^CD200^+^CD43^−^CD103^−^CD11C^−^CD25^−^CD38^−^CD138^−^Kappa^+^Lambda^−^，免疫球蛋白轻链Kappa限制性表达，提示为单克隆B细胞。骨髓染色体核型：44～46,XX,del(6q21),del(7q22),del(19q12),+M1～M3[cp5]/46,XX。骨髓细胞基因重排：IGH FR1-JH、IGH FR2-JH、IGK VK-JK重排阳性。骨髓病理：考虑淋巴组织增殖性疾病；骨髓免疫组化：CD20^+^PAX5^+^、CD5^+^、CD10^−^、BCL6^−^、MUM1^+^、BCL2^+^、CD138部分^+^、VS38C部分^+^、λ^−^、κ^+^、CyclinD1^−^、CD23^−^、Ki67<5％^+^、EBER^−^、MPO粒系^+^、CD42b巨核^+^、CD235红系^+^、CD34血管^+^、CD117肥大细胞^+^，结合免疫组化，符合具有浆样分化的低级别B细胞淋巴瘤累及骨髓，考虑为淋巴浆细胞淋巴瘤。骨髓单克隆淋巴细胞二代测序检测到以下基因突变：TNFAIP3（29.55％）、CCND3（1.92％）、CXCR4（8.93％）、ATM（25.81％），未检测到MYD88突变。肠系膜血管CTA增强：小肠黏膜弥漫性水肿，回肠黏膜空肠化改变，考虑血液系统疾病累及可能。PET-CT：纵隔区淋巴结代谢指标稍高，最大标准摄取值（SUVmax）为3.2。综上，诊断为MYD88^WT^CXCR4^MUT^华氏巨球蛋白血症（WM），华氏巨球蛋白血症国际预后评分（IPSS WM）3分，高危组。

患者反复腹泻，体质差，且患者家属强烈拒绝化疗。2021年4月19日起给予泽布替尼治疗，同时予以间断血浆置换，患者腹泻无好转，IgM下降但很快回升。考虑泽布替尼单用效果不佳，加用硼替佐米1.6 mg/m^2^，每周1次皮下注射，共3周。患者腹泻仍无好转，IgM无下降。停用泽布替尼及硼替佐米，应用BR（苯达莫司汀+利妥昔单抗）方案治疗。治疗1周后患者腹泻次数减少，2周后大便次数正常，2个疗程后IgM正常，腹部增强CT示小肠壁未见明显增厚。3个疗程后免疫固定电泳阴性，评估疗效为完全缓解。BR方案巩固治疗3个疗程后应用奥妥珠单抗单抗维持，每3个月1次，共8次。随访至2024年3月，患者仍持续完全缓解中。

